# The association between academic self-esteem and college students’ positive psychological qualities: the mediating role of general self-efficacy

**DOI:** 10.3389/fpsyg.2026.1788167

**Published:** 2026-04-22

**Authors:** Jing Li, Xianghui Yan

**Affiliations:** 1 Anhui Water Conservancy Technical College, Hefei, Anhui, China; 2 Department of Education and Psychology, Southwest Minzu University, Chengdu, Sichuan, China

**Keywords:** academic self-esteem, college students, mediating role, positive psychological qualities, self-efficacy

## Abstract

**Background:**

Previous studies have primarily focused on the various psychological factors influencing college students’ well-being, yet research on the specific role of academic self-esteem in fostering positive psychological qualities remains limited, especially when considering the mediating effect of general self-efficacy. Existing relevant research has only verified the potential mediating role of general self-efficacy in similar psychological construct relationships, failing to explicitly identify the specific type of mediation or quantify the relative contributions of direct and indirect effects. Meanwhile, empirical evidence for this association is scarce within the cultural and educational context of Chinese college students. This study aims to explore the association between academic self-esteem and college students’ positive psychological qualities, and to examine the mediating role of general self-efficacy, identifying its specific mediation type and quantifying the contribution ratios of direct and indirect effects.

**Methodology:**

The study was conducted online among students from six universities and vocational colleges in Anhui, Zhejiang, and Sichuan Provinces of China, with a total of 1,025 participants completing self-reported electronic questionnaires. After strict screening of valid responses, 940 valid questionnaires were collected. Mediation analysis was performed using the PROCESS macro (Model 4) with the bias-corrected percentile Bootstrap method to verify the specific mediation type and quantify the effect size of general self-efficacy in the relationship between academic self-esteem and positive psychological qualities.

**Results:**

Showed that academic self-esteem was significantly and positively associated with positive psychological qualities (*p* < 0.001), and general self-efficacy played a partial mediating role between them [*β* = 0.37, 95% CI = (0.28, 0.38), *p* < 0.01]. The indirect effect of academic self-esteem on positive psychological qualities through general self-efficacy accounted for 51.85% of the total effect, while the direct effect accounted for 48.15%.

**Conclusion:**

Enhancing college students’ academic self-esteem and general self-efficacy not only promotes their positive psychological qualities more effectively but also improves their mental health. This study makes three notable contributions to the existing literature. First, it explicitly identifies the mediating role of general self-efficacy between academic self-esteem and positive psychological qualities as partial complementary mediation, addressing the deficiency of previous research that only verified the existence of a mediating effect without identifying its specific type. Second, it quantifies the relative contribution ratios of direct and indirect effects, achieving an in-depth decomposition of the statistical mediation pathway. Third, it empirically validates the dual pathway of direct prediction and indirect mediation through general self-efficacy among Chinese undergraduate and vocational college students, enriching cross-cultural empirical research on positive psychological quality cultivation and providing a more precise theoretical basis for targeted psychological interventions.

## 1 Introduction

In the realm of higher education, college students face numerous challenges that test not only their academic capabilities but also their psychological resilience. Psychological constructs such as academic self-esteem and general self-efficacy are of paramount importance in shaping their well-being and life success. Academic self-esteem refers to an individual’s evaluation of their abilities and worth within an academic context, playing a critical role in motivating students, sustaining their perseverance, and enhancing overall academic performance (Vaingankar et al., 2022). In contrast, general self-efficacy is defined as the belief in one’s capacity to execute actions necessary to achieve desired outcomes; it is a broad psychological trait that transcends specific domains and plays a pervasive influence on various aspects of life (Bong and Skaalvik, 2003; Chung et al., 2020; Colom et al., 2019).

Extensive research has been conducted on the relationship between academic self-esteem and positive psychological qualities such as resilience, optimism, and overall life satisfaction (Wang D. et al., 2017; Noguri et al., 2022), and these studies have consistently confirmed a significant positive correlation between the two. According to the Broaden-and-Build Theory proposed by Fredrickson (2001), positive emotions can expand an individual’s cognitive and behavioral scope, thereby accumulating psychological resources (such as resilience and optimism). As a stable source of positive academic emotions, the role of academic self-esteem in promoting positive psychological qualities is essentially a specific manifestation of this theory in the academic field. For instance, high levels of academic self-esteem have been found to enhance students’ adaptability and subjective well-being (Stein et al., 2007; Mazereel et al., 2021). The Reciprocal-Effects Model (REM) proposed by Marsh and Craven (2006) points out that there is a mutually reinforcing relationship between academic self-concept and academic performance. As a core dimension of academic self-concept, the role of academic self-esteem in promoting positive psychological qualities is universal across contexts (such as sports and health), which provides core theoretical support for this study’s hypothesis that “academic self-esteem positively predicts positive psychological qualities.”

However, the underlying pathway of this relationship remains relatively unexplored, and general self-efficacy may serve as a potential mediating factor. In recent years, researchers have begun to investigate the mediating role of general self-efficacy across different contexts (Smith et al., 2013; Weiner et al., 2012), suggesting that it may act as a crucial link between academic self-esteem and positive psychological qualities. A study by Park and Peterson (2010) emphasized that self-efficacy not only was associated with academic performance but also shapes individuals’ psychological resilience when facing life challenges. Despite these findings, existing research still has obvious gaps and deficiencies, particularly within the cultural and educational context of China, especially in Anhui, Zhejiang, and Sichuan Provinces, which typify the development characteristics of central, eastern coastal, and western China, respectively. Most studies only verify the superficial correlation between variables but fail to deeply explore the underlying mediation mechanism; empirical evidence is mostly based on samples from top universities in first-tier cities, lacking targeted research on college students in central and western China, vocational college students, and rural college students who account for a large proportion of the population in the three provinces.

**Figure 1:**
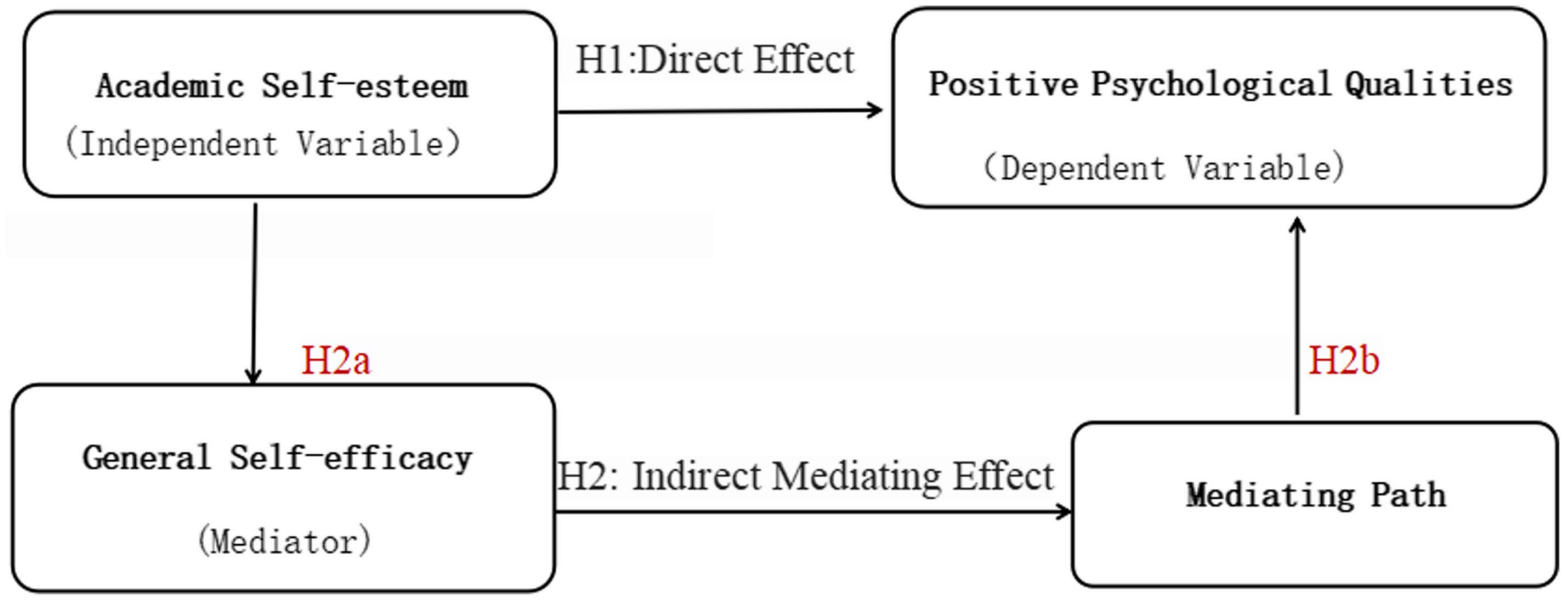
Hypothetical theoretical model of the relationships among academic self-esteem, general self-efficacy, and positive psychological qualities. This model depicts the proposed relationships among the key variables: academic self-esteem as the independent variable, positive psychological qualities as the dependent variable, and general self-efficacy as the mediating variable. H1 posits a direct positive predictive effect of academic self-esteem on positive psychological qualities. H2 posits a partial mediating effect of general self-efficacy, with H2a representing the path from academic self-esteem to general self-efficacy, and H2b representing the path from general self-efficacy to positive psychological qualities. The model is visually represented with academic self-esteem on the left, general self-efficacy in the middle, and positive psychological qualities on the right; arrows indicate the proposed directional associations (H1: direct arrow from academic self-esteem to positive psychological qualities; H2a: arrow from academic self-esteem to general self-efficacy; H2b: arrow from general self-efficacy to positive psychological qualities).

Therefore, this study aims to explore the complex and dynamic relationships among college students’ academic self-esteem, general self-efficacy, and positive psychological qualities. By analyzing the interactions among these variables, the study elucidates the mediating role of general self-efficacy in promoting positive psychological qualities, thereby providing new theoretical insights and empirical support for enhancing college students’ mental health and academic success. The findings of this study will contribute to the development of more effective intervention strategies to foster the comprehensive academic and personal development of college students. The hypothetical model (Figure 1) concisely summarizes the core theoretical framework of the study and lays a foundation for the subsequent empirical testing of the proposed relationships.

In addition to these individual-level factors, parental rearing style represents an important environmental antecedent that shapes students’ self-perception development. According to Baumrind’s parenting style theory, authoritative parenting—characterized by warmth and appropriate autonomy-granting—fosters adolescents’ self-esteem and efficacy beliefs, while authoritarian parenting tends to undermine these psychological resources (Baumrind, 1991). As parenting styles precede and influence the formation of academic self-esteem and self-efficacy during development (Bandura, 1977), it is theoretically warranted to control for their effects when examining the unique contributions of these constructs to positive psychological qualities.

### 1.1 The relationship between academic self-esteem and the positive psychological qualities

Positive psychological qualities refer to enduring individual traits that shape cognitive perspectives, emotional experiences, and coping strategies. These qualities reflect a proactive and optimistic mindset, serve as the foundation of inner psychological strength, stimulate human potential, and enhance psychological resilience to effectively resist mental illnesses. Furthermore, they promote personal and social progress and contribute to a happier life (Chang et al., 2022; Delle Fave et al., 2017; Wong et al., 2010; Wang R. A. H. et al., 2017). In a systematic review of positive mental health in young people, Vaingankar et al. (2022) identified self-efficacy, interpersonal competence, and personal growth as its core dimensions, and confirmed that academic-related self-perception is a key antecedent influencing these dimensions. The conceptualization of positive psychological qualities in this study is grounded in this review, ensuring the scientific validity and comprehensiveness of the variable definition.

College students face multiple sources of pressure that may lead to anxiety and depression, making positive psychological qualities crucial for maintaining their psychological well-being. The increasing prevalence of mental health issues among college students underscores the urgency of exploring pathways to promote positive psychological qualities (Lasheras et al., 2020; Solla Montero and Morales-Rodríguez, 2021; Pei and Wang, 2022).

Academic self-esteem refers to a student’s evaluation and perception of their academic abilities, achievements, and value. It involves their sense of worth and confidence in academic pursuits, and their willingness to tackle challenges and persist through difficulties. Academic self-esteem is closely linked to motivation, engagement, and success in educational settings (Urbańska-Grosz et al., 2023). Existing empirical research has consistently found a significant negative association between college students’ academic self-esteem scores and their levels of depression and anxiety, indicating that low academic self-esteem increases students’ vulnerability to maladaptive psychological states and emotional disorders (Jalene et al., 2019; Melton et al., 2016; Kenney et al., 2015). In contrast, high academic self-esteem can buffer the negative effects of academic pressure on mental health.

Despite these findings, critical research gaps remain. While prior studies have established a basic positive association between academic self-esteem and attributes such as resilience and optimism (Mazereel et al., 2021; Vaingankar et al., 2022), several limitations hinder a comprehensive understanding of this relationship (Guo et al., 2013; Stein et al., 2007; Mazereel et al., 2021). First, most studies only verify surface-level correlations rather than empirically testing the direct predictive effect of academic self-esteem on positive psychological qualities, leaving the causal direction and intensity unclear. Second, relevant research is predominantly based on Western cultural and educational contexts, and the applicability of these conclusions to the Chinese context—where higher education places extreme emphasis on academic performance and students face unique academic and social pressures—lacks sufficient empirical validation. Third, existing studies rarely distinguish between different sub-groups of college students (e.g., vocational vs. undergraduate, rural vs. urban), leading to a lack of targeted insights for specific student populations.

From the perspective of positive psychology and social cognitive theory, academic self-esteem, as a positive academic self-evaluation, can shape students’ proactive cognitive appraisals and behavioral tendencies in learning and life. High academic self-esteem fosters a sense of academic competence, which in turn enhances the willingness to engage in challenges and the ability to cope with setbacks—both core components of positive psychological qualities. Based on this theoretical elaboration and in response to the identified research gaps, the first hypothesis is proposed:

H1: Academic self-esteem significantly and positively predicts positive psychological qualities.

### 1.2 The mediating role of general self-efficacy

General self-efficacy, originally conceptualized by Bandura (1977) within social cognitive theory, refers to an individual’s belief in their capacity to execute actions necessary to achieve desired outcomes. Bandura proposed that self-efficacy beliefs originate from four major sources of information, including performance achievements and vicarious experiences, and play a central role in the regulation of motivation and behavior. Schwarzer further operationalized this construct as an individual’s overall confidence in coping with various challenging situations or new tasks, developing the General Self-Efficacy Scale (GSES) that has been widely used in research and applied settings.

Empirical evidence supports these theoretical propositions. Studies have shown that general self-efficacy is a significant predictor of various positive psychological outcomes, including resilience, optimism, and life satisfaction (Gokbayrak, 2016; Vaingankar et al., 2022). Individuals with higher general self-efficacy are better equipped to manage stress and adapt to changing circumstances, contributing to positive psychological states. Caprara and Steca (2005) further verified that self-efficacy beliefs, as key determinants of prosocial behavior, promote life satisfaction across different age groups, aligning with the positive association between general self-efficacy and positive psychological qualities examined in this study. Moreover, research indicates that academic self-esteem positively correlates with enhanced self-efficacy beliefs (Denkinger et al., 2021; Noguri et al., 2022).

Despite this evidence, the existing literature has notable deficiencies in exploring the mediating pathway between academic self-esteem and positive psychological qualities. First, most studies only verify the existence of the mediating effect of general self-efficacy but fail to explicitly identify the specific type of mediation (e.g., partial vs. full mediation), leaving the nature of this mediating link unclarified. Second, few studies have quantified the relative contribution of the indirect effect compared to the direct effect, making it impossible to accurately grasp the strength of the mediating pathway. Third, the underlying psychological process by which academic self-esteem shapes general self-efficacy and further promotes positive psychological qualities has not been deeply elaborated; existing research only provides a superficial description without revealing the internal cognitive and emotional transmission process.

To address these research gaps, it is essential to establish a theoretical foundation for understanding how academic self-esteem contributes to positive psychological qualities. Social cognitive theory offers a robust framework for elucidating this mediating process. According to Bandura (1977), self-efficacy beliefs are cultivated through mastery experiences, with academic self-esteem representing the cognitive internalization of such experiences within the educational domain. Students with high academic self-esteem internalize their academic achievements, thereby strengthening their general self-efficacy—the cross-situational belief in one’s capacity to succeed across various life domains. Once established, this generalized efficacy functions as a cognitive resource that shapes individuals’ perceptions, interpretations, and responses to challenges. Students possessing heightened general self-efficacy are more inclined to set challenging goals, employ effective coping strategies, and recover from setbacks—all characteristic of positive psychological qualities such as optimism, resilience, and perseverance. Empirical evidence substantiates this theoretical pathway: Chemers et al. (2001) demonstrated that academic self-efficacy not only predicts academic performance but also significantly enhances students’ personal adjustment and stress management (Li et al., 2018; Okonofua et al., 2016), thereby fostering positive psychological outcomes. Consequently, general self-efficacy serves as a pivotal mechanism that translates domain-specific academic confidence into generalized psychological resilience, underscoring its potential as a target for interventions designed to enhance student well-being and success (Denkinger et al., 2021; Vaingankar et al., 2022; Morales-Rodríguez et al., 2019; Ghay et al., 2022).

Based on the above theoretical foundation, empirical evidence, and identified research gaps, the second hypothesis is proposed:

H2: General self-efficacy plays at least a partial mediating role in the relationship between academic self-esteem and college students’ positive psychological qualities.

### 1.3 Hypothetical theoretical model

A hypothetical theoretical model depicting the direct and indirect relationships among the variables (academic self-esteem as the independent variable, positive psychological qualities as the dependent variable, and general self-efficacy as the mediating variable) is presented in Figure 1. This model visually synthesizes the proposed relationships of the study, including the direct predictive effect of academic self-esteem on positive psychological qualities and the indirect mediating pathway through general self-efficacy.

## 2 Methods

### 2.1 Participants

This study adopted a stratified random sampling method stratified by school type (undergraduate institutions/higher vocational colleges) and grade (freshman to senior) to ensure the representativeness and validity of the research sample.

Inclusion criteria: (1) Full-time undergraduate or vocational college students enrolled in six selected institutions in Anhui, Sichuan, and Zhejiang Provinces; (2) Voluntary participation with the ability to understand Chinese and complete the electronic questionnaire independently; (3) Clear understanding of the research purpose and provision of informed consent.

Exclusion criteria: (1) Incomplete questionnaire responses (with more than 5% of scale items unanswered); (2) Random or repetitive responses (e.g., selecting the same option for all items in a scale); (3) Non-full-time students or students from non-target institutions; (4) Participants who withdrew their informed consent after completing the questionnaire.

This study was approved by the Ethics Committee of Anhui Water Conservancy Technical College (Ethics Approval No.: AWCTC-2024-035), and all research procedures strictly complied with the Ethical Guidelines for Psychological Research Involving Human Participants formulated by the Chinese Psychological Society. Before the survey, the informed consent form was presented as the first page of the electronic questionnaire, which included the research purpose, data collection content, anonymous processing of data, voluntary participation and withdrawal rights, and research team contact information. Participants could only access the formal questionnaire after clicking “I agree.”

Participants were recruited through collaboration with faculty administrators at each institution, who distributed the online questionnaire link to class WeChat groups. All responses were anonymous and voluntary. A total of 1,025 students were initially surveyed, and after screening according to the above criteria, 940 valid questionnaires were finally collected, with a valid response rate of 91.71%. Among the valid participants, 51.70% were male and 48.30% were female; the average age was 19.85 years (SD = 1.53); 31.70% were freshmen, 27.55% were sophomores, 20.85% were juniors, and 19.80% were seniors. In terms of place of origin, 24.26% were from urban areas, 21.49% from townships, and 54.26% from rural areas. Regarding parental rearing style, 15.43% were authoritarian, 80.64% were democratic, and 3.94% were indulgent.

The sample of this study has good representativeness of the target population (full-time undergraduate and vocational college students in Anhui, Sichuan, and Zhejiang Provinces). First, the stratified random sampling method based on school type and grade ensures that the sample structure is consistent with the overall distribution of higher education students in the three provinces, avoiding sampling bias. Second, the six selected institutions cover comprehensive universities, local undergraduate colleges, and vocational technical colleges, matching the main types of higher education institutions in the target regions. Third, the demographic characteristics of the sample (gender ratio, age distribution, grade structure, rural–urban origin proportion, parental rearing style) are highly consistent with the published statistical data of college students in the three provinces, and the valid sample size (940) is sufficient to support the generalizability of the research conclusions to the target population.

### 2.2 Measures

#### 2.2.1 The demographic measures

Demographic measurement indicators included age, gender, grade, place of origin, type of school, parental rearing styles, and parents’ education level. Age required participants to fill in their specific age numerically. Gender was measured with a binary option (male/female). Grade was classified into four levels: freshman, sophomore, junior, and senior. School type was divided into undergraduate institutions and higher vocational (technical) colleges. Place of origin was categorized as urban, township, and rural. Parental rearing style was classified as authoritarian, democratic, and indulgent. Parental education level (for both father and mother) was divided into five levels: uneducated, primary school, middle school/technical secondary school, junior college, and undergraduate or above. Except for age, all other indicators were measured using single-choice questions, requiring participants to select the option that best matched their actual situation.

#### 2.2.2 The Chinese college student positive psychological quality scale

This scale was developed by Wanjin Meng and Qun Guan, consisting of 62 items grouped into six dimensions: Wisdom and Knowledge, Courage, Human Nature (Emotion), Justice, Temperance, and Transcendence. These six dimensions are designed to assess 20 positive psychological qualities. Sample items include: “I usually maintain a positive attitude when facing challenges” and “I am good at learning from failures” etc. The scale employs a 5-point Likert rating system, where 1 signifies “very unlike me” and 5 denotes “very like me.” A higher score in each dimension indicates a better development of the corresponding positive psychological quality among college students.

Reliability and Validity Justification: (1) Reliability: Cronbach’s *α* coefficient was used to test internal consistency (a standard method for multi-item Likert scales). The standardized Cronbach’s α coefficient of the scale was 0.986, with all dimension α coefficients exceeding 0.89, far above the 0.7 threshold, indicating excellent reliability; (2) Validity: Confirmatory Factor Analysis (CFA) was used to test construct validity, aligning with the core need to verify the scale’s theoretical structure. Fit indices (χ^2^/df = 2.35, CFI = 0.92, RMSEA = 0.06) met standards, demonstrating good construct validity. In this study, the standardized Cronbach’s alpha coefficient of the scale stood was 0.986, demonstrating high reliability of the data, thus making it suitable for further analysis.

#### 2.2.3 The academic self-esteem scale

This scale was adapted from the Performance State Self-Esteem Scale compiled by Heatherton and Polivy, with a rigorous adaptation and validation process for the Chinese college student sample: (1) Item modification: 5 items irrelevant to the Chinese academic context (e.g., items related to Western extracurricular activities) were revised to align with Chinese college students’ learning scenarios (e.g., “I feel confident in my exam performance” instead of “I feel confident in my club activities”); (2) Expert review: 3 educational psychology experts and 2 college counselors evaluated the relevance and cultural adaptability of the revised items, with 3 items further adjusted based on their feedback to ensure alignment with academic self-esteem connotations; (3) Pre-survey validation: A pre-survey was conducted with 200 college students from the target regions, and item analysis (CITC > 0.4) and exploratory factor analysis (EFA) were used to retain 18 valid items with good discriminability.

The revised scale consists of 18 items, such as “I feel confident in my learning abilities” and “I believe I can achieve good grades.” It adopts a 1–5 scoring method, where 1 means “very inconsistent,” 2 means “basically inconsistent,” 3 means “uncertain,” 4 means “basically consistent,” and 5 means “very consistent.” The higher the individual’s total score, the higher the level of academic self-esteem.

Reliability and Validity Justification: (1) Reliability: Cronbach’s *α* coefficient was adopted, which is highly compatible with the scale’s unidimensional structure, and the result showed an α coefficient of 0.926 (> 0.7), indicating good internal consistency; (2) Validity: Content validity was verified by expert evaluation (scale CVI = 0.92) to ensure items align with the connotation of academic self-esteem; Exploratory Factor Analysis (EFA) was used to test construct validity, meeting the structural verification needs of the revised scale. One common factor was extracted (cumulative variance explanation rate = 68.35%), indicating acceptable construct validity. In this investigation, the Cronbach’s alpha coefficient of the scale stands at 0.926, attesting to the high reliability of the data and its appropriateness for deeper analysis.

#### 2.2.4 The general self-efficacy scale

The General Self-Efficacy Scale originally developed by Ralf Schwarzer et al. and subsequently translated into Chinese by Caikang Wang et al. (2001), has gained widespread usage in domestic academic circles. Its reliability and validity have undergone rigorous verification. Consisting of 10 items, such as “If I try my best, I can always solve the problems.” and “I can handle whatever happens to me with ease.” etc. This scale serves as a one-dimensional assessment instrument primarily designed to evaluate individuals’ confidence in their abilities during stressful situations. It utilizes a 1–4 rating system, wherein 1 signifies “not true at all,” 2 implies “hardly true,” 3 represents “moderately true,” and 4 denotes “exactly true.” A higher total score reflects a stronger overall confidence in coping with diverse environmental challenges (Zhang et al., 2022).

Reliability and Validity Justification: (1) Reliability: Combined Cronbach’s *α* coefficient (0.962 > 0.7) and Corrected Item-Total Correlation (CITC, all > 0.4) for comprehensive verification, meeting the rigorous requirements for unidimensional scale reliability assessment; (2) Validity: Confirmatory factor analysis (CFA) confirmed the unidimensional structure with good fit indices (χ^2^/df = 2.35, CFI = 0.92, RMSEA = 0.06). Convergent validity was supported by a significant positive correlation with the Academic Self-Esteem Scale (*r* = 0.696, *p* < 0.001). Discriminant validity was established as the square root of the average variance extracted (AVE) for each construct exceeded its correlations with other constructs, indicating that the constructs are empirically distinct. In the present study, the Cronbach’s alpha coefficient for the scale stands at 0.962, and all CITC values for the analyzed items exceed 0.4, indicating high data reliability and suitability for further analysis.

#### 2.2.5 Control variables

In this study, several demographic characteristics (gender, age, grade, place of origin, and parental rearing style) were controlled as confounding variables, based on prior research showing their associations with the main study variables. Gender was coded as a dummy variable (1 = male, 2 = female). Age was measured by the number of years. Grade was divided into 4 levels (1 = freshman to 4 = senior). place of origin was divided into 3 levels (1 = urban, 2 = township, 3 = rural). Parental rearing style was measured using a single-choice question with three categories (1 = authoritarian, 2 = democratic, 3 = indulgent).

### 2.3 Data analysis

All data analysis in this study was conducted using IBM SPSS statistics 27.0 and the PROC-ESS macro. Descriptive statistical analysis was used to calculate the mean value, standard deviation, and correlation coefficients of all variables. Harman’s single-factor test was conducted to evaluate whether a common method bias existed in the questionnaire data. The rotated principal component analysis revealed 8 factors with eigenvalues greater than 1. The total variation explained by the first factor was 21.275%, which fell below the critical threshold of 40%, indicating that the study did not exhibit a notable common method bias. Prior to main analyses, regression assumptions were assessed. Normality of residuals was examined using Q-Q plots, which indicated approximately normal distributions. Multicollinearity was evaluated using variance inflation factor (VIF), with all predictor VIF values below 3, suggesting no serious multicollinearity concerns.

Subsequently, Pearson correlation analysis was performed to explore the bivariate relationships among variables and understand the extent of their mutual influence. Finally, the PROCESS macro was employed to investigate the associations of academic self-esteem and general self-efficacy on positive psychological qualities. To ensure the accuracy of the results, including gender, grade, place of origin, and parental rearing style, were controlled in the analysis to more precisely examine the mediating effect. All significance tests were two-tailed, with the significance level set at *p* < 0.05.

## 3 Results

### 3.1 Descriptive statistics and correlation analysis

The mean, standard deviations, Cronbach’s alpha coefficient and correlation coefficients for all variables are shown in Table 1. The bivariate analysis revealed that general self-efficacy was significantly and positively associated with positive psychological qualities (*r* = 0.719, *p* < 0.001) and academic self-esteem (*r* = 0.696, *p* < 0.001); academic self-esteem was positively associated with positive psychological qualities (*r* = 0.675, *p* < 0.001). These findings provide initial support for the subsequent regression and mediation analysis.

**Table 1:** Descriptive Statistics and correlation analysis of variables (*N* = 940).

Variable	M	SD	1	2	3	4	5	6
1. Gender	1.48	0.50						
2. Grade	1.44	0.76	0.15**					
3. Place of origin	2.30	0.83	0.02	-0.04				
4. Parenting style	1.89	0.43	0.03	0.04	0.02			
5. Positive psychological quality	3.51	0.81	−0.16**	−0.08**	−0.07*	0.02		
6. Academic self-esteem	3.18	0.72	−0.21**	−0.12**	−0.05	−0.03	0.68***	
7. General self-efficacy	2.73	0.68	−0.21**	−0.05	−0.12**	0.04	0.72***	0.70***

*p < 0.05; **p < 0.01; ***p < 0.001.

### 3.2 Hypotheses testing

After standardizing all survey questionnaire variables, mediation analysis was conducted using Hayes’ PROCESS macro (Model 4) with 5,000 bootstrap samples and 95% bias-corrected confidence intervals. Covariates including gender, grade, place of origin, and parental rearing style were entered in both the mediator and outcome models (Lau et al., 2017; Powers et al., 2017; Shyr et al., 2022). This analysis focused on exploring the mediating role of general self-efficacy between academic self-esteem and positive psychological qualities. A Bootstrap sampling test with a sampling frequency of 5,000 times was applied to assess this mediating effect. If the 95% confidence interval (CI) excluded 0, the mediating effect path was considered statistically significant; if the 95% CI included 0, the mediating effect path was considered non-significant. To clarify the type of mediation, the definition proposed by Hayes (2013) was adopted: a “partial complementary mediation” exists when both the direct effect of the independent variable on the dependent variable and the indirect effect through the mediator remain statistically significant, and both effects are in the same direction. Detailed results are presented in Table 2.

**Table 2:** Regression analysis of the mediating model of general self-efficacy between academic self-esteem and positive psychological qualities (*N* = 940).

Variable	Positive psychological quality				General self-efficacy				Positive psychological quality			
	B	SE	t	β	B	SE	t	β	B	SE	t	β
Constant	1.23***	0.17	7.32	-	0.84***	0.13	6.42	-	0.75***	0.15	4.85	-
Control variables												
Gender	−0.04	0.04	−0.86	−0.02	−0.09**	0.03	−2.75	−0.07	0.02	0.04	0.42	0.01
Grade	−0.01	0.03	−0.37	−0.01	0.04	0.02	1.66	0.04	−0.03	0.02	−1.25	−0.03
Place of origin	−0.04	0.02	−1.60	−0.04	−0.07***	0.02	−3.60	−0.08	0.00	0.02	0.02	0.00
Parenting style	0.08	0.05	1.59	0.04	0.03	0.04	0.89	0.02	0.06	0.04	1.33	0.03
Independent variable												
Academic self-esteem	0.72***	0.03	24.77	0.64	0.65***	0.02	28.61	0.68	0.35***	0.04	9.74	0.31
Mediator												
General self-efficacy									0.58***	0.04	15.35	0.48
R^2^	0.42				0.50				0.54			
F	133.83***				184.66 ***				178.80 ***			

*p < 0.05; **p < 0.01; ***p < 0.001.

Table 2 shows that the mediation analysis comprises three models, and the empirical results verify the proposed relationships in the hypothetical theoretical model (Figure 1). The regression equations are as follows:

College students’ positive psychological qualities = 1.23–0.04*gender-0.01*grade−0.04*place of origin+0.08*parenting style+0.72*college students’ academic self-esteem;

College students’ general self-efficacy = 0.84–0.09*gender+0.04*grade-0.07*place of origin+0.03*parenting style+0.65*college students’ academic self-esteem;

College students’ positive psychological qualities = 0.75 + 0.02*gender-0.03*grade+0.00*place of origin+0.06*parenting style+0.35*college students’ academic self-esteem+0.58*College students’ general self-efficacy.

Subsequently, the mediating effect test was performed using the bias-corrected nonparametric percentile Bootstrap method. The findings revealed that academic self-esteem was associated with positive psychological qualities through an indirect path: “academic self-esteem ⇒ general self-efficacy ⇒ positive psychological qualities.” The 95% confidence interval determined by Bootstrap sampling excluded zero, and the direct effect of academic self-esteem on positive psychological qualities remained significant [*β* = 0.35, SE = 0.04, 95% CI = (0.28, 0.42), *p* < 0.001] after including general self-efficacy as a mediator. This indicates a partial complementary mediating role of general self-efficacy between academic self-esteem and positive psychological qualities.

**Figure 2:**
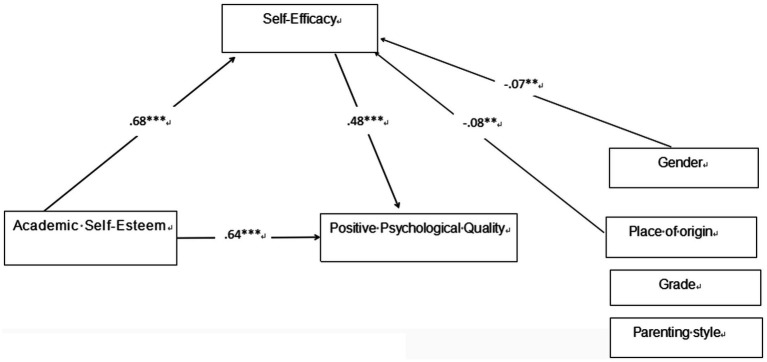
The mediated model. *p* < 0.01; ***p < 0.001.

Specifically, the total effect of academic self-esteem on positive psychological qualities was 0.72 [SE = 0.03, 95% CI = (0.66, 0.78), *p* < 0.001]; the indirect effect through general self-efficacy was 0.37 [SE = 0.02, 95% CI = (0.28, 0.38), *p* < 0.01], accounting for 51.85% of the total effect; the direct effect was 0.35 [SE = 0.04, 95% CI = (0.28, 0.42), *p* < 0.001], accounting for 48.15% of the total effect. Both the direct and indirect effects were in a positive direction, confirming that academic self-esteem promotes positive psychological qualities through dual pathways (direct prediction + indirect mediation via general self-efficacy). Detailed outcomes are shown in Table 3 and Figure 2.

**Table 3:** Summary of mediation test results (*N* = 940).

Pathway	Effect type	Effect value	SE	95% CI	*Z*	% of Total effect
Academic self-esteem= > self-efficacy= > positive psychological quality	Indirect effect	0.37	0.02	[0.28, 0.38]	16.03	51.85%
Academic self-esteem = > self-efficacy	X= > M	0.65	0.02	[0.61, 0.70]	28.61	—
Self-efficacy = > positive psychological quality	M= > Y	0.58	0.04	[0.50, 0.66]	15.35	—
Academic self-esteem= > positive psychological quality	Direct effect	0.35	0.04	[0.28, 0.42]	9.74	48.15%
Academic self-esteem = > positive psychological quality	Total effect	0.72	0.03	[0.66, 0.78]	24.77	100%

*p < 0.05; **p < 0.01; ***p < 0.001.

Bootstrap samples = 5,000; CI = bias-corrected 95% confidence interval. All effects are standardized coefficients (β). *p* < 0.001 for all effects.

## 4 Discussion

### 4.1 Result analysis

To the best of our knowledge, this study contributes to the existing literature by examining the mediating role of general self-efficacy and the pathway through which academic self-esteem relates to positive psychological qualities, and the empirical results provide empirical support for the rationality of the hypothetical theoretical model (Figure 1) proposed in this study. Notably, this study explicitly identifies the mediation type as partial complementary mediation and quantifies the relative contribution of direct and indirect effects—an improvement over previous research that only verified the existence of mediation without in-depth effect decomposition (Weiner et al., 2012; Yimer et al., 2022).

Previous research has paid insufficient attention to the direct relationship between academic self-esteem and positive psychological qualities, yet scholars have emphasized that academic self-esteem enhances college students’ academic achievements (Noguri et al., 2022; Mazereel et al., 2021; Stein et al., 2007), and a strong positive relationship exists between academic performance and self-esteem (Stein et al., 2007; Mazereel et al., 2021; Urbańska-Grosz et al., 2023). This study confirms a significant positive predictive association of academic self-esteem on positive psychological qualities, validating Hypothesis 1. The underlying reasons for this direct effect can be explained from three aspects:

First, college students with a high level of academic self-esteem hold a more optimistic and affirming stance toward their learning abilities and academic achievements. This positive mindset fuels their learning motivation and self-confidence, enabling them to concentrate better on learning tasks and actively confront academic challenges. This proactive approach undoubtedly fosters the cultivation of their positive psychological qualities, such as perseverance and optimism.

Second, college students endowed with high academic self-esteem are more likely to derive a sense of fulfillment and accomplishment from their studies. When they achieve good grades or master new knowledge through their own efforts, they perceive personal growth and progress, which generates positive psychological experiences. These experiences not only enhance their self-confidence but also stimulate their creativity and exploratory motivation, further promoting the development of positive psychological qualities.

Third, college students with high academic self-esteem have a stronger ability to cope with setbacks and failures in learning. They have enough confidence in their own abilities and believe that they can overcome difficulties and achieve success through effort. This perseverance in the face of challenges is an important part of positive psychological qualities. When faced with academic difficulties, they are more inclined to take active problem-solving strategies rather than give up easily, which helps them better adapt to various challenges in learning and life.

The results indicate that academic self-esteem holds a key position in individuals’ psychological development. It not only was directly associated with positive psychological qualities but also shows a significant indirect effect through the important mediating factor of general self-efficacy, validating Hypothesis 2. Existing research has confirmed a notable association between self-efficacy and learning engagement (Wang et al., 2016; Ugarte-Gil et al., 2023; Urbańska-Grosz et al., 2023), and higher learning engagement makes it easier for students to attain academic success, forming a positive feedback loop. Specifically, students with high levels of academic self-esteem tend to have a more confident perception of their learning abilities and achievements. This positive self-evaluation implicitly boosts their general self-efficacy.

The high self-efficacy fostered by elevated academic self-esteem further acts as a potent impetus for the emergence and enhancement of positive psychological qualities. This is due to the intimate connection between general self-efficacy and an individual’s mental state. People with high general self-efficacy tend to exhibit superior mental health, manifesting as few negative emotions such as depression and anxiety (Görgülü et al., 2021; Torniainen-Holm et al., 2016). Individuals with high self-efficacy can assess their own abilities and the pressures they face more objectively and rationally (Krueger et al., 2011; Jo et al., 2024). As a result, they can effectively adjust their mindset, actively respond to challenges, and take appropriate measures to solve problems, thereby maintaining psychological stability. Meanwhile, numerous studies have also confirmed that general self-efficacy plays an important role in enhancing individuals’ happiness (Wolf et al., 2020; Arora et al., 2016). There is an association between positive psychological qualities and self-efficacy, and the development of students’ positive psychological qualities can be achieved by improving their self-efficacy levels. As an emotional experience, general self-efficacy is instrumental in mitigating negative emotions. It also serves as a motivator for individuals to adhere to healthy behaviors, thus contributing to psychological stability. Moreover, a higher level of self-efficacy instills greater confidence in overcoming life’s obstacles, further promoting the cultivation of positive psychological traits (Shin and Choi, 2019; Jaarsma et al., 2017). In essence, self-efficacy is not only intricately linked to positive psychological qualities but also plays a pivotal role in enhancing individuals’ inner senses of joy, happiness, and control.

These findings align with Bandura’s social cognitive theory, which posits that self-efficacy beliefs mediate the relationship between prior performance experiences and subsequent behavioral outcomes. Academic self-esteem, as the cognitive residue of past academic achievements, provides the foundational mastery experiences that nourish general self-efficacy. This generalized efficacy belief system then becomes the psychological engine driving positive qualities such as optimism, resilience, and perseverance. The mediation identified in this study thus represents not merely a statistical association but a theoretically coherent cognitive pathway: academic self-esteem → generalized confidence → positive psychological functioning.

In conclusion, individuals with high self-efficacy have a stronger sense of control over their lives, are more easily satisfied, and thus experience a higher level of happiness. They bravely face difficulties, are willing to accept challenges, and demonstrate greater persistence and perseverance in the learning process. This positive attitude and action reflect positive psychological qualities such as optimism, resilience, and self-regulation. Therefore, it is clear that academic self-esteem, by enhancing self-efficacy, promotes the development of positive psychological qualities like confidence, optimism, and resilience.

### 4.2 Practical implications

The practical implications of understanding the associations between academic self-esteem and college students’ positive psychological qualities, particularly through the mediating role of general self-efficacy, are manifold. Educational institutions can leverage these insights to design and implement interventions that enhance both academic self-esteem and general self-efficacy, thereby promoting students’ overall psychological well-being. For instance, schools and universities might develop programs focused on building students’ competencies and confidence in their academic abilities through personalized feedback, mentorship, and skills development workshops. By fostering an environment that emphasizes mastery and personal growth rather than solely performance-based outcomes, educators can help students internalize a more robust sense of self-worth in academic settings.

Additionally, integrating self-efficacy training into existing curricula can empower students to translate academic confidence into broader life skills, thereby improving resilience, adaptability, and stress management. Duan et al. (2024) found that emotional regulation self-efficacy mediates the relationship between college students’ perceived social support and positive psychological qualities, suggesting that intervention programs should combine academic self-esteem enhancement with emotional regulation training—for example, guiding students to use positive academic experiences to regulate negative emotions, which can further strengthen the effect of promoting positive psychological qualities. Practical strategies could include goal-setting workshops, peer-supported learning initiatives, and the incorporation of real-world problem-solving activities that highlight the applicability of academic skills to everyday challenges. Through these initiatives, students can develop a more generalized sense of efficacy, enhancing their ability to face diverse challenges beyond the academic sphere. Furthermore, by recognizing the interconnectedness of academic self-esteem and general self-efficacy, educational policymakers and administrators are better positioned to advocate for systemic changes that prioritize mental health resources and support systems, ultimately fostering a more holistic educational experience that nurtures both intellectual and emotional capacities.

### 4.3 Limitations and prospects

While this study offers valuable insights into the associations among academic self-esteem, general self-efficacy, and positive psychological qualities in college students, several limitations should be noted. First, due to the cross-sectional design, the mediation examined in this study is statistical rather than causal. The proposed pathways represent associative relationships consistent with theoretical predictions, but causal inferences cannot be drawn. Second, the findings are situated within the cultural and educational context of Chinese higher education, where collectivist values and academic emphasis may shape self-esteem and self-efficacy. Thus, generalizability to other countries or educational systems with different cultural norms is limited. Third, the use of self-reported measures may introduce biases such as social desirability or inaccurate self-assessment. Fourth, other potential mediating or moderating variables (e.g., personality traits, social support) were not fully accounted for in the current model. Fifth, although Harman’s single-factor test indicated no severe common method bias, this method has inherent limitations.

Future research should employ longitudinal or experimental designs to establish temporal precedence and causality. Expanding studies to diverse cultural contexts and employing mixed-methods approaches would further enrich understanding of how these psychological constructs interact. To strengthen control over common method bias, future studies should adopt more rigorous statistical approaches, such as the unmeasured latent method factor technique or marker variables, in addition to procedural remedies (e.g., participant anonymity, counterbalanced item order, and validated scales). Interdisciplinary approaches integrating psychology, educational theory, and sociology may also inform more comprehensive intervention strategies to support student well-being.

## 5 Conclusion

In summary, the association between academic self-esteem and positive psychological qualities is mediated by general self-efficacy. The indirect and direct effects accounted for 51.85 and 48.15% of the total effect, respectively, indicating that academic self-esteem is significantly associated with positive psychological qualities both directly and indirectly through general self-efficacy. Therefore, for college students with lower positive psychological qualities, enhancing their academic self-esteem and general self-efficacy can more effectively foster the development of these qualities. Theoretically, this study clarifies the mediating pathway between academic self-esteem and positive psychological qualities, enriches the empirical basis of positive psychology in Chinese higher education, and validates the applicability of Bandura’s social cognitive theory in this context. Practically, it provides actionable insights for colleges and universities: targeted interventions (e.g., academic feedback, self-efficacy training) can be designed to improve students’ psychological well-being, and local education departments can reference the findings to optimize mental health resource allocation, especially for vulnerable groups such as vocational college students and rural students.

## Data Availability

The raw data supporting the conclusions of this article will be made available by the authors, without undue reservation.
